# Refractory pneumonia due to persistent SARS-CoV-2 infection in an immunocompromised host successfully treated with extended course nirmatrelvir/ritonavir

**DOI:** 10.1128/asmcr.00049-24

**Published:** 2025-01-21

**Authors:** Shiro Sonoda, Sho Shimada, Atsushi Sawada, Tomoka Yasuda, Tsuyoshi Shirai, Haruhiko Furusawa, Yukie Tanaka, Kousuke Tanimoto, Hiroaki Takeuchi, Yasunari Miyazaki

**Affiliations:** 1 Department of Respiratory Medicine, Institute of Science Tokyo, Tokyo, Japan; 2 Department of Molecular Microbiology and Immunology, Graduate School of Medical and Dental Sciences, Institute of Science Tokyo, Tokyo, Japan; 3 Department of High-risk Infectious Disease Control, Institute of Science Tokyo, Tokyo, Japan; 4 Institute of Science Tokyo, Center for Infectious Disease Education and Analysis (TCIDEA), Tokyo, Japan; Rush University Medical Center, Chicago, Illinois, USA

**Keywords:** SARS-CoV-2, follicular lymphoma, XBB, XBB.2.3, IgG

## Abstract

**Background:**

Immunocompromised individuals are infected with persistent severe acute respiratory syndrome coronavirus 2 (SARS-CoV-2) and often refractory to treatment.

**Case Summary:**

A 56-year-old woman undergoing chemotherapy for follicular lymphoma experienced relapsing pulmonary infiltrates and a consistently low cycle threshold value of nasal swab SARS-CoV-2 PCR over a 5-month period. Despite repeated remdesivir and glucocorticoid therapy, her respiratory symptoms worsened and progressed to respiratory failure. Viral genome analysis revealed that the variant strain at onset was XBB, whereas the strains repeatedly detected more than 2 months after onset were XBB.2.3.

**Conclusion:**

The genome analysis indicated that genomic mutations accumulate due to continuous XBB infection. The patient recovered after treatment with nirmatrelvir/ritonavir for 20 days.

## INTRODUCTION

Persistent severe acute respiratory syndrome coronavirus 2 (SARS-CoV-2) infections sporadically reported in individuals with immunocompromising conditions, such as B-cell deficiencies, HIV, or those who have undergone solid-organ transplants, have emerged as a significant clinical challenge. Persistent SARS-CoV-2 infection has been defined as SARS-CoV-2 infection meeting one of the following criteria > 30 days after onset: presence of virus, as determined by isolation, genomic, or equivalent tests; and accumulation of genetic changes in the virus or the presence of ongoing symptoms (1). Treatments for persistent SARS-CoV-2 infection have not yet been established, making clinical management challenging (2). Moreover, the emergence of viral variants has reduced the efficacy of monoclonal antibodies, restricting therapeutic choices to antiviral agents (3).

The present report describes a patient with relapsing refractory SARS-CoV-2 pneumonia who was successfully treated with extended use of nirmatrelvir/ritonavir (4, 5). Viral genomic analysis of multiple samples obtained during the course of disease showed that the XBB variant was detected at disease onset, whereas the XBB.2.3 variant was detected continuously thereafter, suggesting continuous infection causing accumulated genomic mutations.

## CASE PRESENTATION

A 56-year-old woman presented with fever, fatigue, and cough in June 2023. At that time, she was receiving second-line treatment with obinutuzumab and bendamustine for follicular lymphoma. Her history included three doses of SARS-CoV-2 vaccine. SARS-CoV-2 PCR testing using nasal swab at her initial visit showed a low cycle threshold (Ct) value (24.7), and a chest X-ray showed bilateral peripheral ground glass opacity, confirming a diagnosis of corona virus disease 2019 (COVID-19) pneumonia. She was hospitalized and treated with remdesivir for 5 days, resulting in symptom improvement and her subsequent discharge from the hospital.

One month later, however, she complained of persistent fever and cough. A chest X-ray in July 2023 showed the appearance of migratory lung infiltrates, and blood tests showed an elevated C-reactive protein concentration (see supplemental Fig. S1 at https://figshare.com/s/8d10c6d8fac67b0f5873?file=51621377). Treatment with levofloxacin for 1 week showed no improvement in infiltrates. At this point, no further investigations were conducted, and 15 mg/day of prednisolone was initiated based on the assumption of secondary organizing pneumonia following COVID-19. Despite a mild improvement in cough, she continued to have a fever and a worsening dyspnea on exertion. Her prednisolone dosage was increased to 25 mg/day, but her dyspnea and pulmonary infiltrates worsened. Nasal swab SARS-CoV-2 PCR in September 2023 showed a low Ct value (22.8), reconfirming COVID-19. She was treated with remdesivir for 5 days and 500 mg/day methylprednisolone for 3 days, followed by discharge from the hospital with 15 mg/day oral prednisolone.

**Fig 1:**
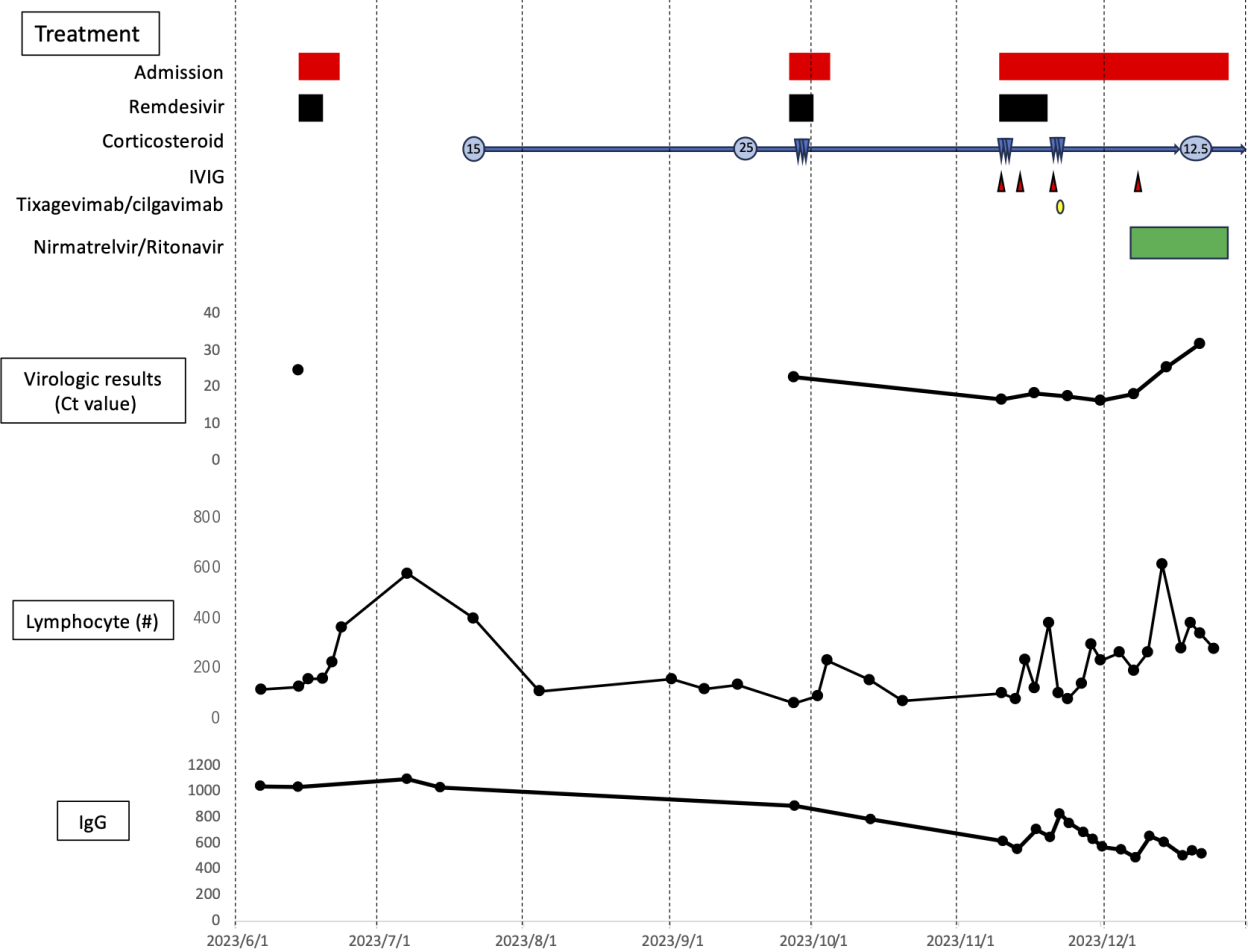
Clinical course of treatment, virologic results, lymphocyte counts, and serum IgG concentrations in the present patient. Treatment: red bars, three cycles of remdesivir, continuous corticosteroid, four cycles of venoglubulin, one cycle of tixagevimab/cilgavimab and 20 days of nirmatrelvir/ritonavir. Numbers within circles indicate daily doses of prednisolones, and blue triangles indicate steroid pulse therapy. Virologic results; filled symbols indicate Ct values of the SARS-CoV-2 PCR assays of nasopharyngeal swabs, with higher Ct values indicating lower viral loads. Samples in June and September were obtained when symptoms appeared, and samples in November were taken approximately once per week. Lymphocytes, IgG; samples were taken during outpatient visits and at admission to hospital, as appropriate.

During tapering of prednisolone over 2 months, however, the patient again experienced dyspnea requiring supplemental oxygen, with a relapse of infiltrates. Nasal swab SARS-CoV-2 PCR in November 2023 showed a very low Ct value (16.7), leading to her readmission for COVID-19 pneumonia. Because her IgG level at admission was low (Fig. 1), she was treated with remdesivir for 10 days and 1000 mg/day methylprednisolone for 3 days, along with intravenous immunoglobulin. In addition, despite reduced efficacy being reported against the variants circulating at the time, tixagevimab/cilgavimab was given for its potential neutralizing antibody effect (6).

Her symptoms and pulmonary infiltrates, however, did not improve, and nasal swab SARS-CoV-2 PCR showed a consistently low Ct value, indicating a poor response to treatment. Her treatment was switched to nirmatrelvir/ritonavir in addition to 25 mg/day maintenance prednisolone, allowing her to be weaned off supplemental oxygen within 7 days. In addition, the SARS-CoV-2 viral load decreased (Fig. 1), and lung infiltrates improved significantly. Finally, the patient completed an extended 20-day course of nirmatrelvir/ritonavir with no adverse effects and was discharged. Nasal swab SARS-CoV-2 PCR was negative 2 months after discharge.

### Viral genome analysis

Viral genome sequences were analyzed in the nasal swab samples obtained from this patient at four time points: 14 June, 27 September, 10 November, and 17 November. Viral RNA was extracted using QIAamp Viral RNA Mini Kits (Qiagen, Venlo, Netherlands), and whole viral genome libraries were prepared with QIAseq SARS-CoV-2 Primer Panel Kits (Qiagen). The resulting viruses were sequenced using the MiSeq Sequencing System (Illumina, San Diego, CA, USA), and viral variants were determined using the CLC Genomics Workbench Software (Qiagen). Genetic analysis of the four SARS-CoV-2 variants, TMDU_No625 (GISAID accession no. EPI_ISL_18866523), TMDU_No626 (GISAID accession no. EPI_ISL_18866564), TMDU_No627 (GISAID accession no. EPI_ISL_18866565), and TMDU_No628 (GISAID accession no. EPI_ISL_18866566), indicated that this patient was initially infected with an XBB variant (TMDU_No625), followed by separate and persistent infection with an XBB.2.3 variant (TMDU_No626, TMDU_No627, and TMDU_No628) (see supplemental Fig. S2 at https://figshare.com/s/8d10c6d8fac67b0f5873?file=51621377). Although the latter three viruses shared the same variant, mutations were identified (see supplemental Fig. S3 at https://figshare.com/s/8d10c6d8fac67b0f5873?file=51621377). There is no genetic mutation associated with resistance to nirmatrelvir.

## DISCUSSION

Persistent SARS-CoV-2 infection has been reported frequently in immunocompromised patients, particularly those with hematological disorders. Its clinical manifestations can vary, ranging from asymptomatic infection to worsening infection with recurrent pneumonia, as in the present patient (7). The incidence of persistent SARS-CoV-2 infection may be underestimated both because its diagnostic criteria have not been definitely established, and because its symptoms vary widely. Some patients may be diagnosed with long COVID, which can also show diverse symptoms. Persistent infection was identified in the present patient following the detection of repeated pulmonary infiltrates that developed after the completion of COVID-19 pneumonia treatment.

Analysis of the viral genome in the four samples obtained at different time points revealed that the patient was initially infected with the XBB variant TMDU_No625, whereas the three other samples collected afterward were XBB.2.3 variants (TMDU_No626, TMDU_No627, and TMDU_No628). We speculate that the most likely scenario is that the XBB variant infected in June was cleared, and reinfection by XBB.2.3 occurred, progressing to persistent infection. Another possibility is that the XBB initially infecting the patient in June mutated and subsequently developed into a persistent infection. The results of the genome-based phylogenetic analysis also indicated the emergence of a genomic mutation designated as XBB.2.3 during the persistent infection period. These findings are consistent with previous studies of persistent SARS-CoV-2 infection, in which genomic mutations were observed in the host (8). There was no evidence of newly acquired drug-resistance mutations during the clinical course of this patient.

Several factors may have contributed to the development of persistent SARS-CoV-2 infection in this patient. First, she was being treated for follicular lymphoma with the anti-CD20 antibody obinutuzumab and the alkylating agent bendamustine just before the onset of COVID-19. Although her IgG level was maintained at the onset of COVID-19, it began to decline during the persistent infection period, suggesting the development of a B-cell deficiency. Obinutuzumab and bendamustine are both known to cause prolonged B-cell dysfunction, even after the termination of treatment, findings consistent with the course of disease in this patient. In addition, prolonged use of prednisolone prior to developing persistent infection may have suppressed B-cell function and reduced viral clearance (9), and the prolonged steroid use in the absence of concurrent antiviral therapy as treatment for organizing pneumonia may also have been a factor as a contributor to ongoing viral replication (10).

Treatment for persistent SARS-CoV-2 infection has yet to be established. Although several case reports have described the effectiveness of off-label treatment of extended nirmatrelvir/ritonavir, alone or in combination with remdesivir against persistent SARS-CoV-2, especially in immunocompromised patients (1, 11), these regimens have not been evaluated enough in clinical trials. As monoclonal antibodies are not expected to be effective against current variants of SARS-CoV-2, antiviral agents may be the only treatment options. The identification of drug-resistant mutations by genomic analysis may help predict the efficacy of antiviral drugs. Although treatment with nirmatrelvir/ritonavir for 5 days has not been approved in Japan, findings in this patient highlight the need to evaluate extended nirmatrelvir/ritonavir treatment of immunocompromised patients with persistent SARS-CoV-2 infection. Extended nirmatrelvir/ritonavir treatment may be a treatment option in patients with persistent infection of SARS-CoV-2 variants resistant to remdesivir.
